# Supplementary material for: Increased mitochondrial calcium levels associated with neuronal death in a mouse model of Alzheimer’s disease
Source: Nat Commun. 2020 May 1;11:2146. doi: 10.1038/s41467-020-16074-2 (PMC7195480; doi:10.1038/s41467-020-16074-2)
Supplement: Supplementary file 1 — Supplementary Information [file 41467_2020_16074_MOESM1_ESM.pdf]

# Supplementary Information

# Increased mitochondrial calcium levels associated with neuronal death in a mouse model of Alzheimer's disease

Calvo-Rodriguez et al.

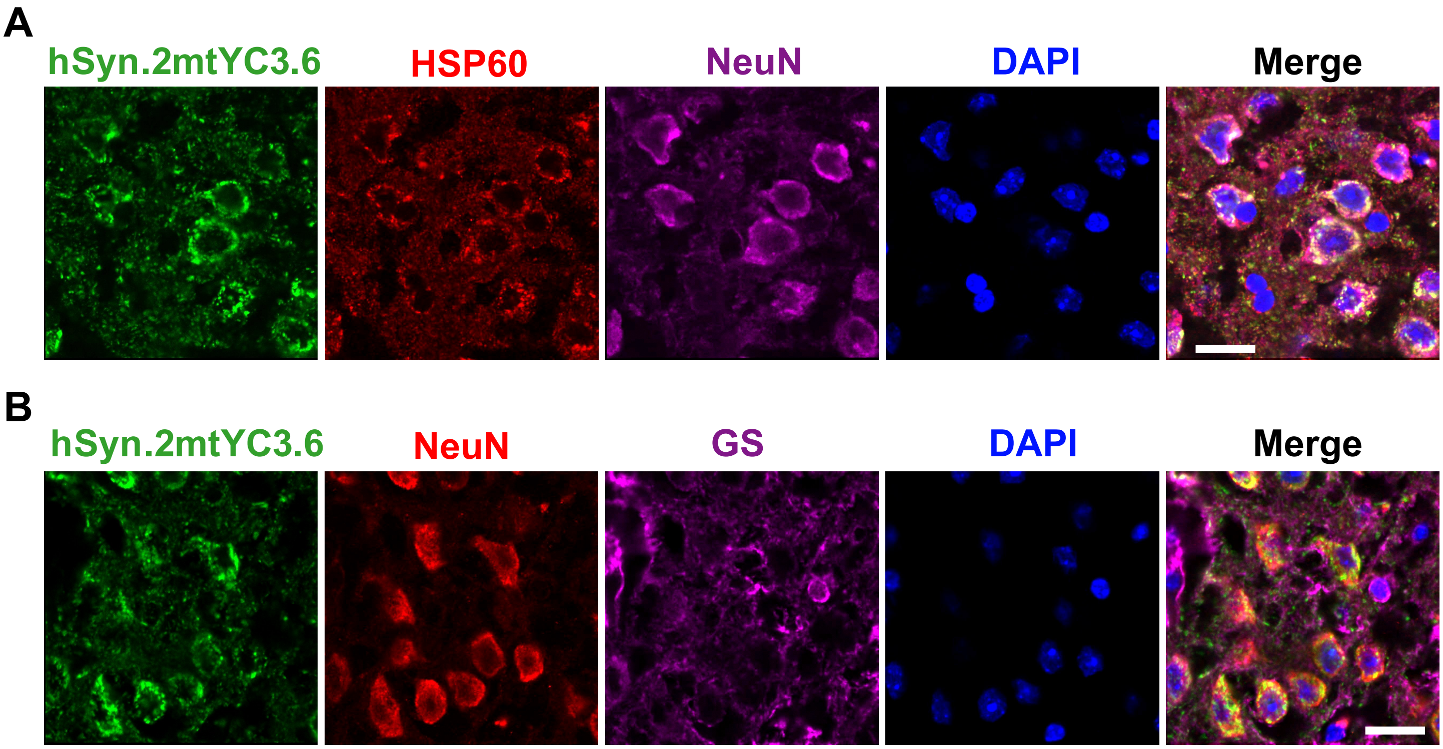


**Supplementary Figure 1. Related to Figure 1. Validation of pAAV.hSyn.2mtYC3.6 *ex vivo*. A.** Colocalization of AAV.hSyn.2mtYC3.6 (green), HSP60 (red) targeting mitochondria and NeuN (magenta) targeting neurons in the cortex shown by immunohistochemistry. Scale bar 20 µm. **B.** Colocalization of AAV.hSyn.2mtYC3.6 (green), NeuN (red) to targeting neurons and GS (magenta) targeting astrocytes in cortex shown by immunohistochemistry. Note that AAV.hSyn.2mtYC3.6 colocalizes with neuronal mitochondria and not with mitochondria in astrocytes. Scale bar 20 µm.

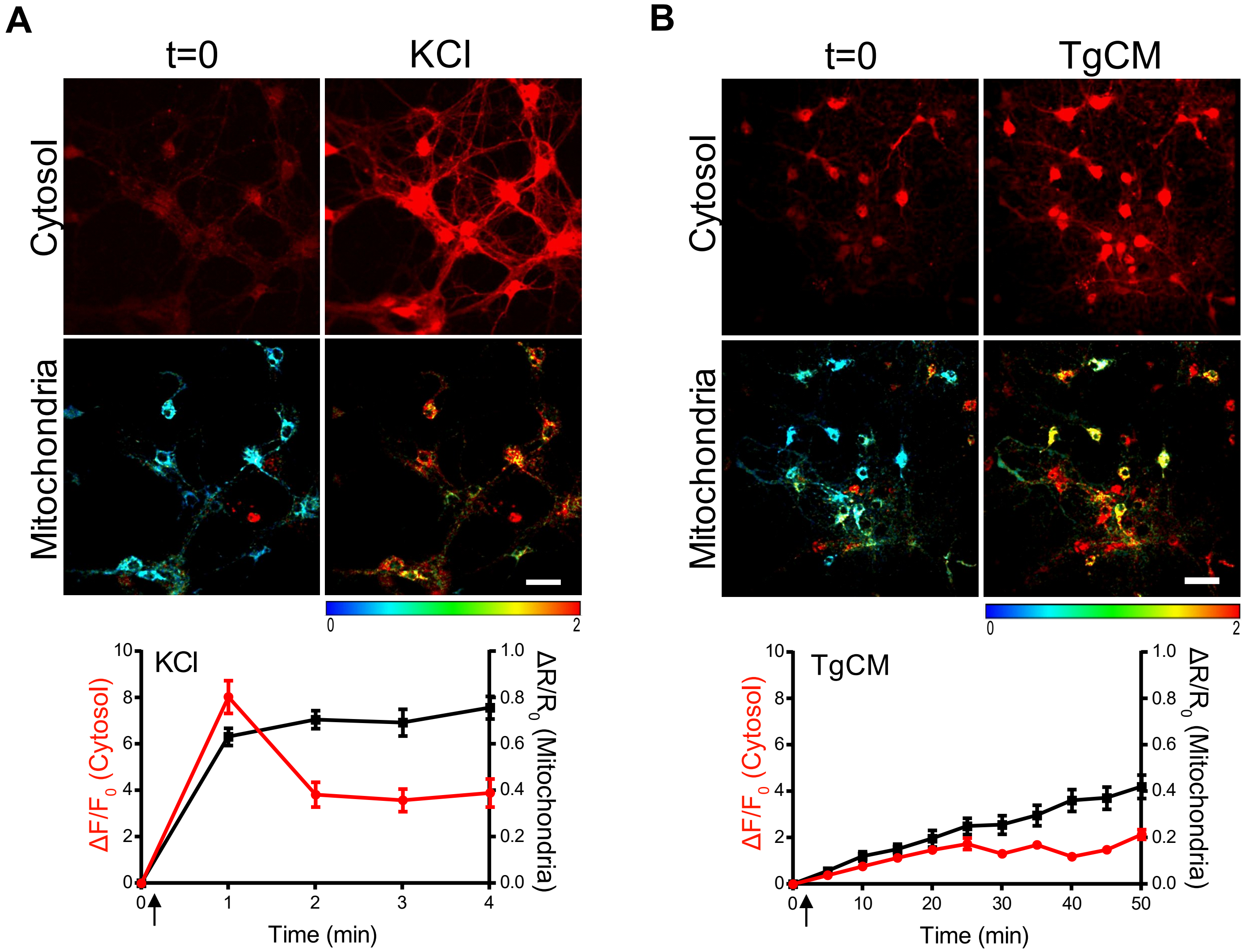


**Supplementary Figure 2. Related to Figure 1. Mitochondrial and cytosolic $Ca^{2+}$ exhibit different dynamics after application of KCl or TgCM.** Calbryte 590 loaded primary neurons expressing hsyn.2mtYC3.6 were used to simultaneously record $[Ca^{2+}]_{cyt}$ (red traces) and $[Ca^{2+}]_{mit}$ (black traces) in response to cell stimulation with either KCl (50 mM) (**A**) or TgCM (**B**). Images (top) show the same field of view before (t=0) and after application of the stimulus in cytosol and mitochondria. Scale bar 20 µm. Changes were plotted together for comparison (bottom). It can be observed that there is a synchronous transient increase of $[Ca^{2+}]_{mit}$ and $[Ca^{2+}]_{cyt}$ after KCl is applied, but the mitochondrial $Ca^{2+}$ extrusion system acts slower than cytosolic $Ca^{2+}$ clearance. On the other hand, after TgCM application, mitochondria acts as a buffer of $[Ca^{2+}]_{cyt}$ increase. Traces are representative for 2-3 independent experiments.

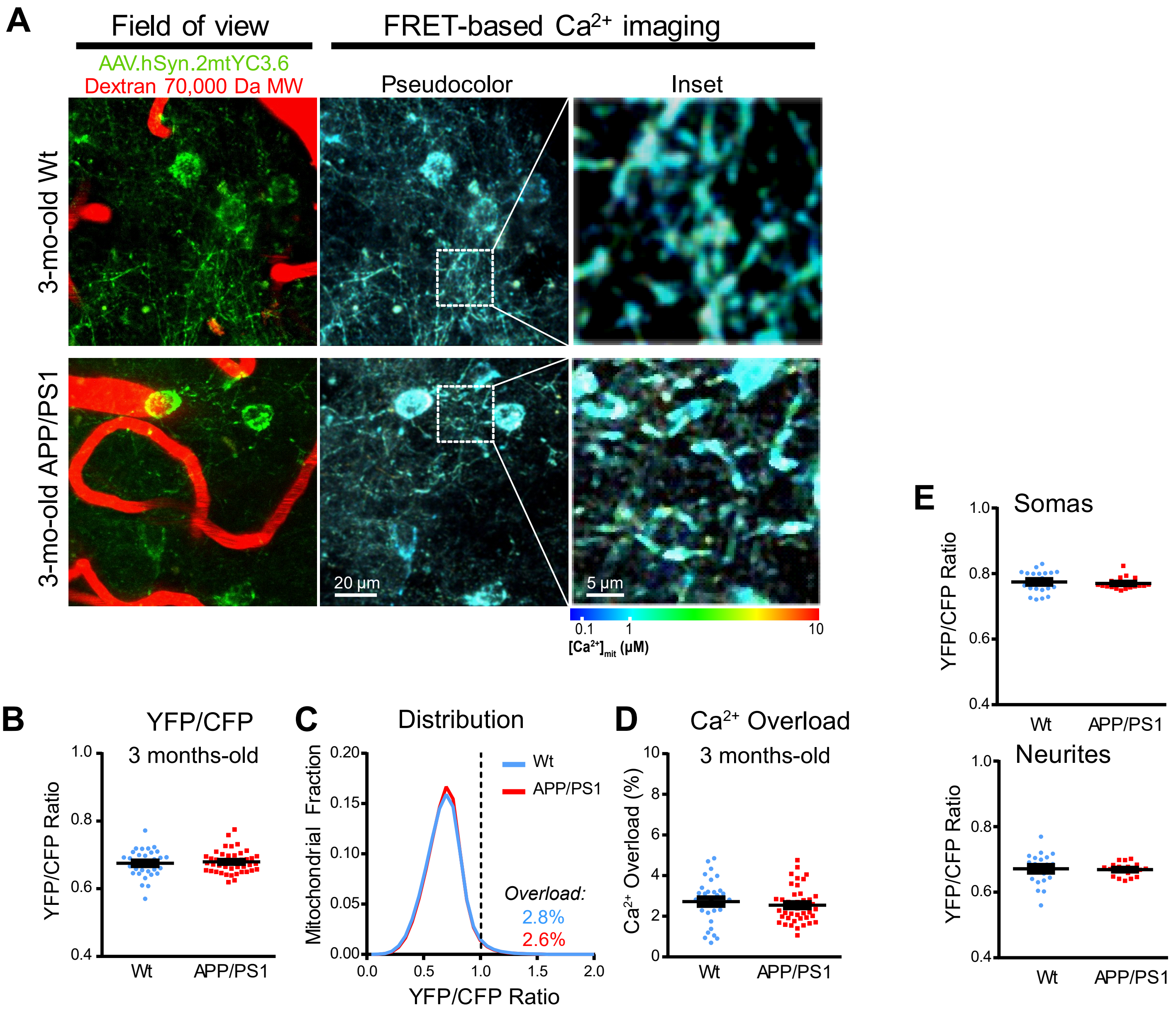


**Supplementary Figure 3. Related to Figure 2. Mitochondrial $Ca^{2+}$ is not altered prior to plaque deposition in APP/PS1 Tg mice. A.** *In vivo* multiphoton microscopy images of neurites and cell bodies expressing AAV.hSyn.2mtYC3.6 in mitochondria in 3-mo-old Wt (top) and 3-mo-old APP/PS1 Tg mice (bottom). FRET-based $Ca^{2+}$ imaging shows pseudocolored images of the field of view and the inset from the white box. Scale bar 20µm and inset 5µm. **B.** 2.5-3-mo-old APP/PS1 Tg mice failed to present mitochondrial $Ca^{2+}$ overload when compared to 2.5-3-mo-old Wt mice. Individual mitochondria were automatically selected for measuring YFP/CFP ratio. Scatter dot plot represents the YFP/CFP ratio in every volume acquired. Error bars represent mean ± SEM. Not-significant (0.67±0.011 (~472 nM $[Ca^{2+}]_{mit}$), n=32 z-stacks from 5 Wt mice, and 0.68±0.010 (~520 nM $[Ca^{2+}]_{mit}$), n=44 z-stacks from 6 APP/PS1 Tg mice. **C.** Histogram of $[Ca^{2+}]_{mit}$ frequency distribution (YFP/CFP ratio). The black dotted line corresponds to 2 SD above the mean $[Ca^{2+}]_{mit}$ in Wt mice (YFP/CFP ratio >1.00, ~1671 nM $[Ca^{2+}]_{mit}$). Ratios above this line were classified as mitochondrial $Ca^{2+}$ overload. **D.** Scatter dot plot represents the percentage of $Ca^{2+}$-overloaded mitochondria in the acquired volumes in 2.5-3-mo-old Wt and 2.5-mo-old APP/PS1 Tg mice. APP/PS1 Tg mice failed to show a significant higher percentage of mitochondria with elevated $Ca^{2+}$. Error bars represent mean ± SEM. Not significant (APP/PS1 Tg mice: 2.59±0.32%, n=44 z-stacks from 6 mice *vs.* Wt mice: 2.80±0.0.37%, n=32 z-stacks from 5 mice). **E.** Comparison of $[Ca^{2+}]_{mit}$ (YFP/CFP ratio) in the different locations (somas and neurites) in 3-mo-old Wt and APP/PS1 Tg mice. Mitochondrial $Ca^{2+}$ levels both in somas and neurites did not differ between Wt and APP/PS1 mice. Error bars represent mean ± SEM. (Somas: 0.77±0.01, n=21 z-stacks from 3 Wt mice, and 0.77±0.003, n=20 z-stacks from 3 APP/PS1 Tg mice. Neurites: 0.67±0.01, n=21 z-stacks from 3 Wt mice, and 0.67±0.004, n=20 z-stacks from 3 APP/PS1 Tg mice).

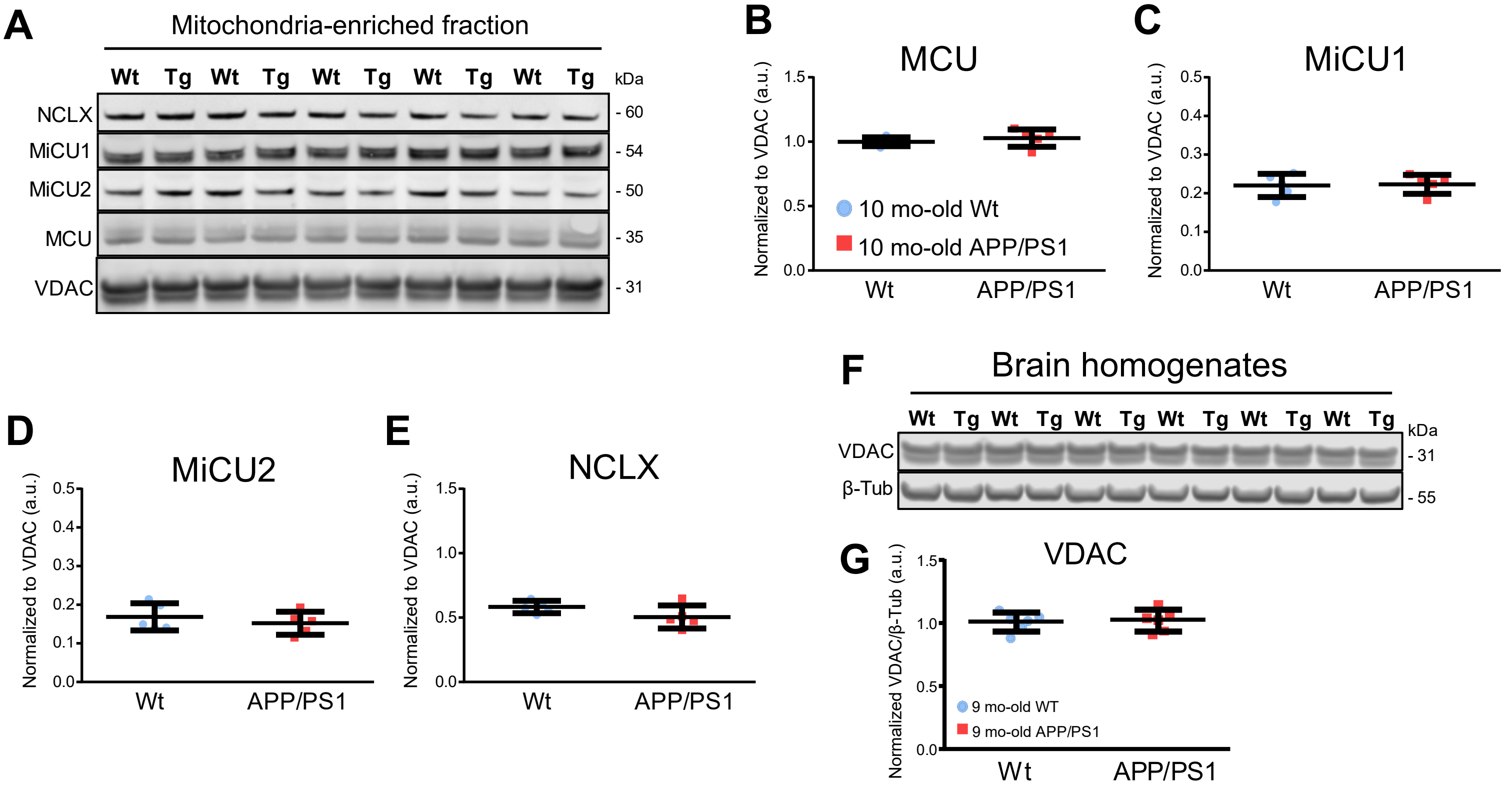


**Supplementary Figure 4. Related to Figure 5. Protein levels of the mitochondrial Ca$^{2+}$ homeostasis are unchanged in the APP/PS1 Tg mouse after plaque deposition. A.** Western blots of the main mitochondrial Ca$^{2+}$ homeostasis proteins (MCU, MiCU1, MiCU2, NCLX) from the mitochondria-enriched fraction of 10-mo-old Wt and APP/PS1 Tg mouse brains (n=5 mice per group). **B-E.** Quantification of the Western blots by densitometry revealed no significant differences between Wt and APP/PS1 Tg mice. **F.** Western blots of the protein used as mitochondrial loading control (VDAC1/porin) on whole brain homogenates from the brain of 10-mo-old Wt and APP/PS1 Tg mice (n=6 mice per group). **G.** Quantification of the Western blots revealed no significant differences between Wt and APP/PS1 Tg mice, confirming its validity as mitochondrial housekeeping loading control.

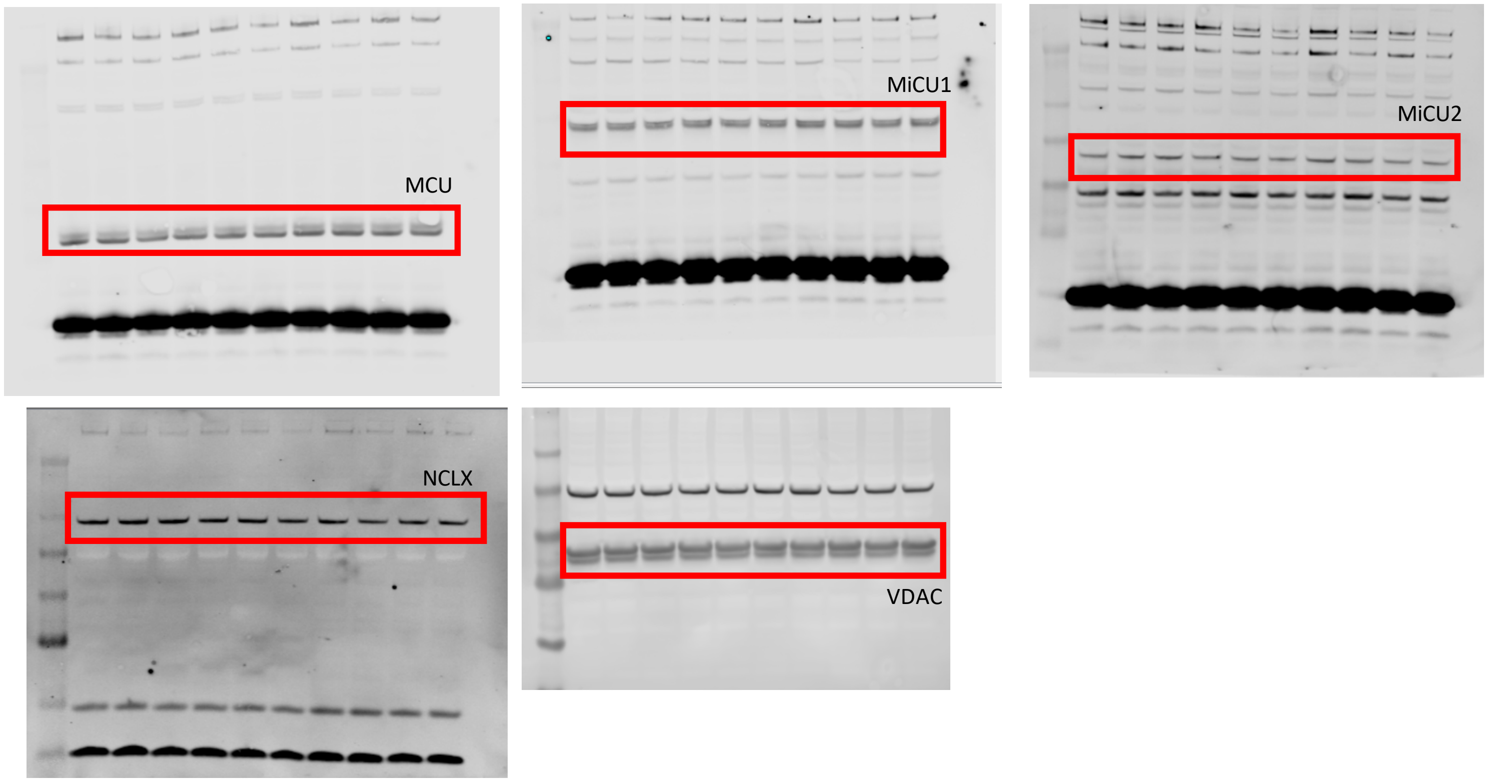

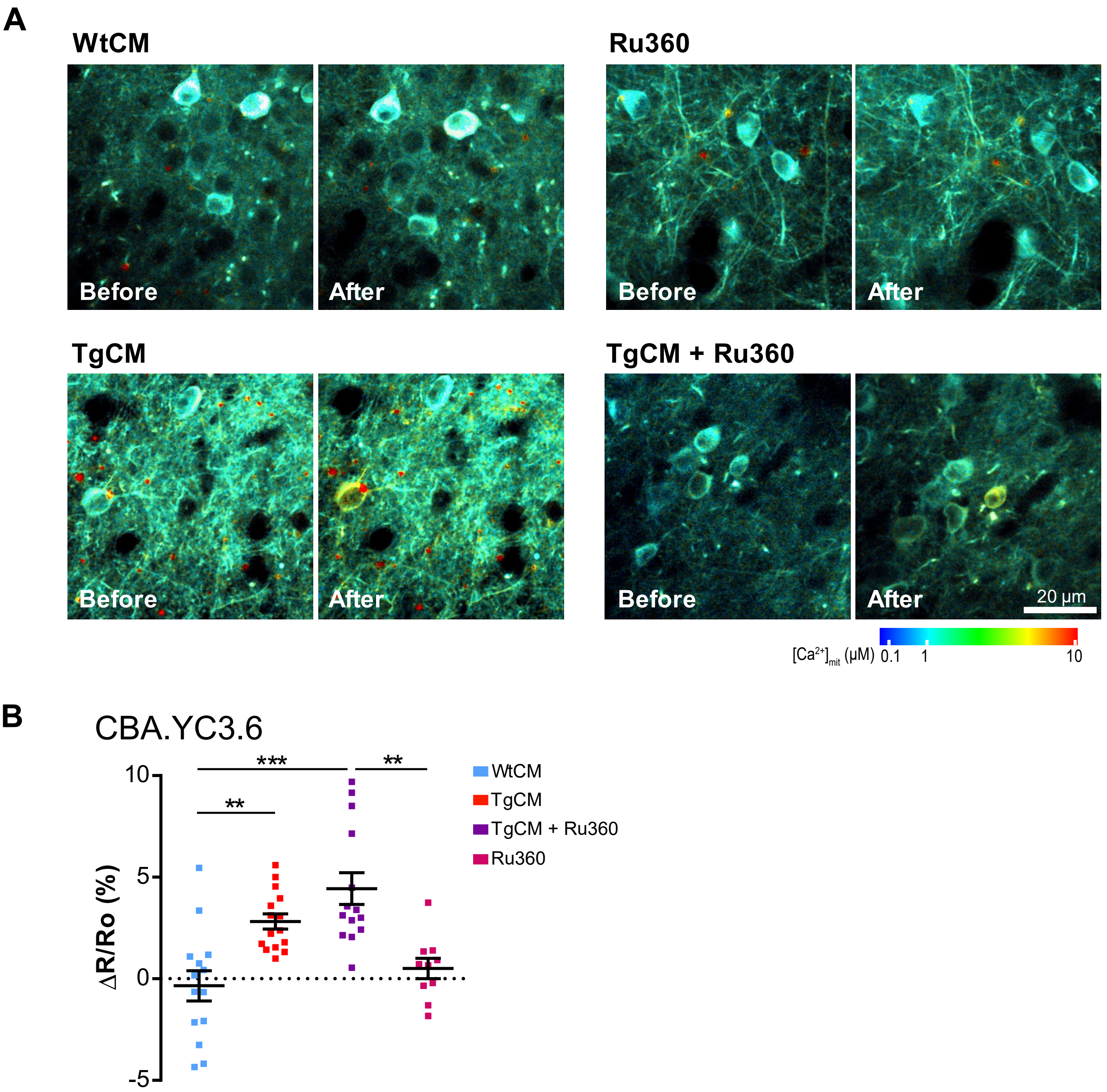


**Supplementary Figure 5. Related to Figure 5. Ru360 does not prevent Aβ-induced cytosolic $Ca^{2+}$ increase *in vivo.*** **A.** Representative pseudocolor pictures of the effects of the MCU complex blocker Ru360 on the increase of $[Ca^{2+}]_{cyt}$ induced by TgCM. TgCM elicited an increase in $[Ca^{2+}]_{cyt}$ even in the presence of Ru360. Scale bar 20µm. **B.** Scatter dot plot represent the relative change in YFP/CFP ratio ($\Delta R/R_0$) for each condition. Bars represent mean ± SEM (-0.34±0.74%, 14 z-stacks WtCM, 2.81±0.37%, 15 z-stacks TgCM; 4.44±0.78%, 14 z-stacks Ru360+TgCM; -0.50±0.50%, 10 z-stacks Ru360 from 2, 3, 3 and 2 mice respectively, ***p*<0.005, *****p*<0.0001).

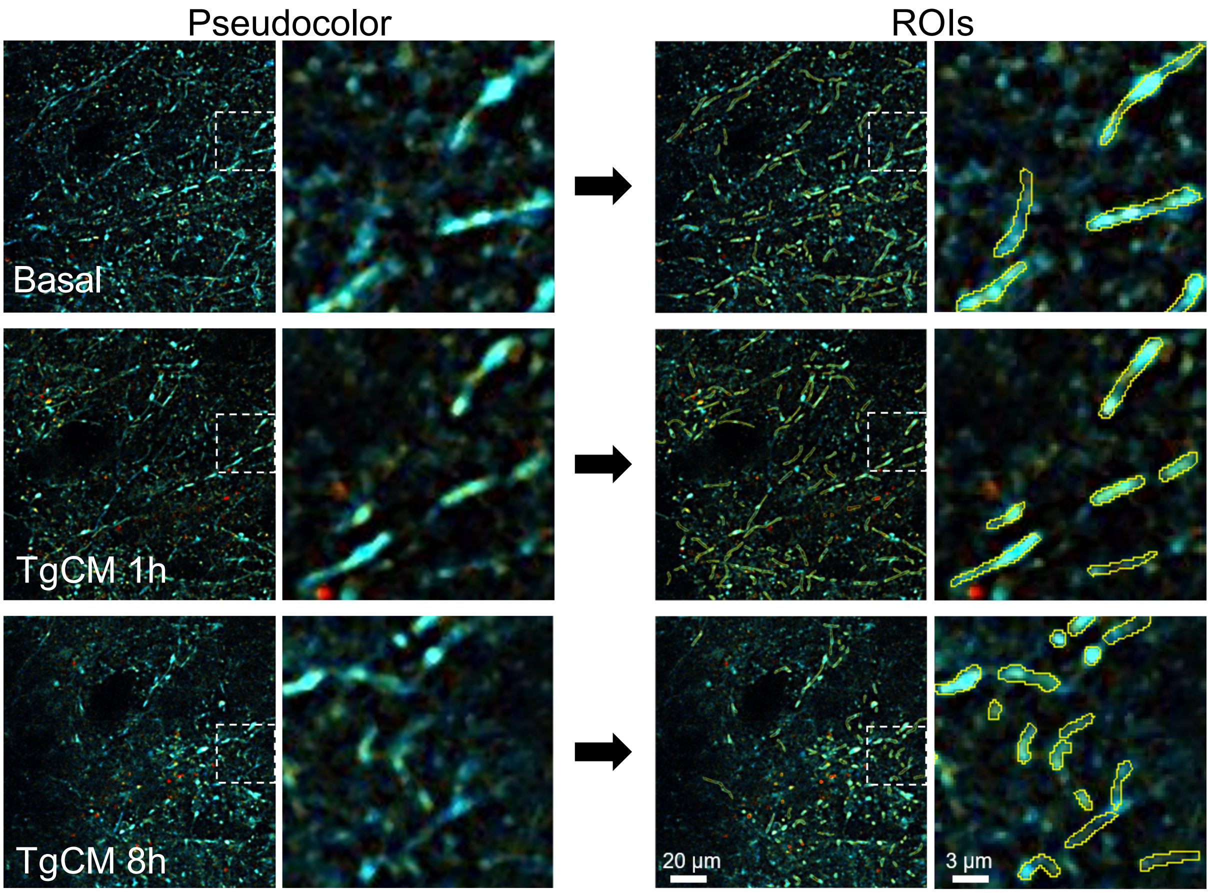


**Supplementary Figure 6. Related to Figure 6. Acquisition of mitochondrial size and shape at different time points.** Pseudocolor images (left) show an example of how ROIs (right) where taken in order to measure area, perimeter and circularity before and after application of TgCM. The same image was selected across the z-stack for the three time points (basal, 1h, 8h) and ROIs were drawn with free hand selection tool in ImageJ around individual mitochondria. 100-125 random individual mitochondria were taken per image. Scale bar 20µm and inset 3µm.

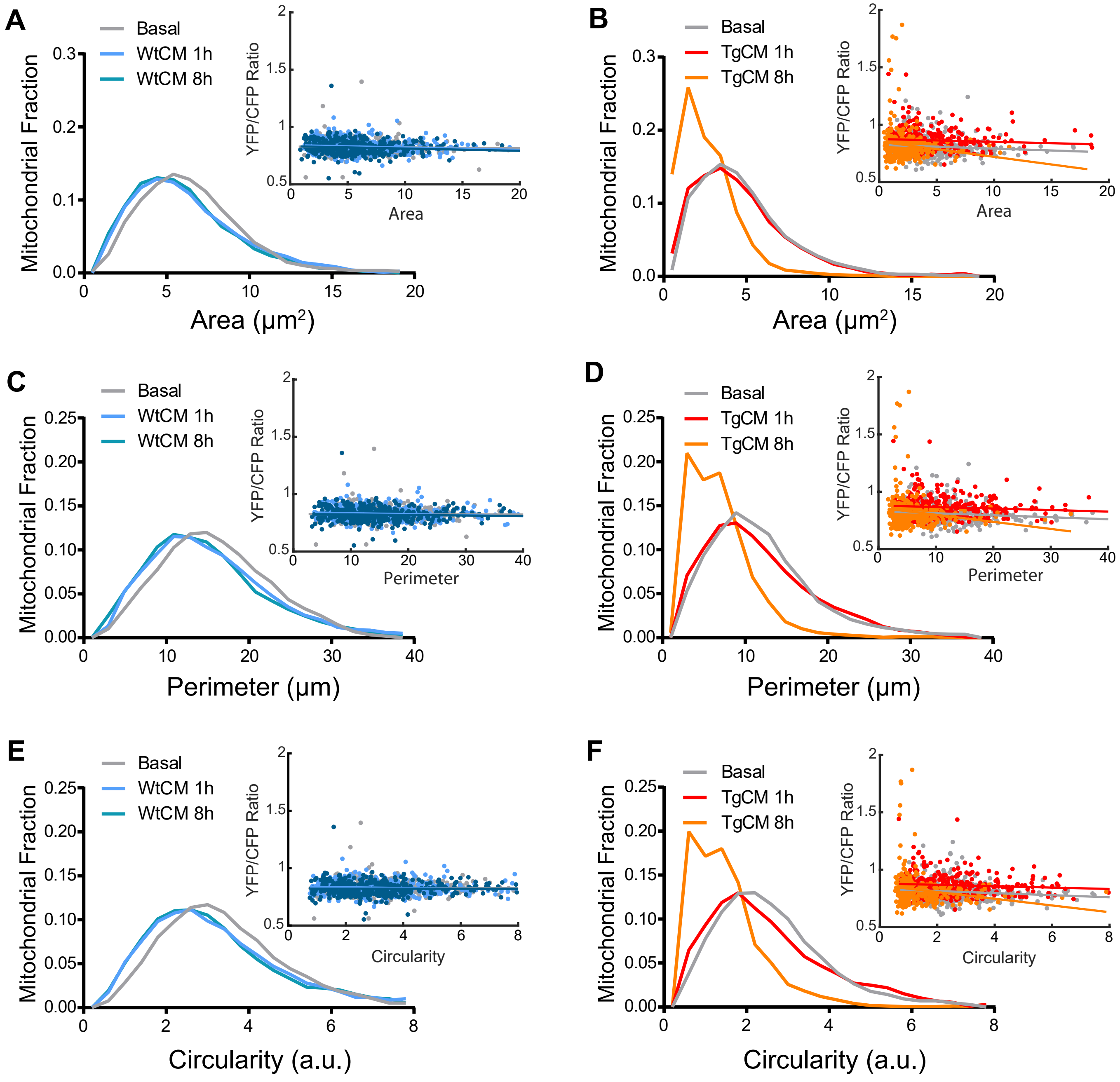


**Supplementary Figure 7. Related to Figure 6. Mitochondrial size and shape are altered 8h after application of TgCM.** Mitochondria area, perimeter and circularity were calculated as a readout of mitochondrial health. The same field of views were analyzed at baseline and at 2 different time points after CM application (1h and 8h). Histograms show mitochondrial fraction distribution for each parameter. Scatter diagrams (insets, top right of every graph) show correlation between $[Ca^{2+}]_{mit}$ (YFP/CFP Ratio) and the corresponding parameter (area, perimeter or circularity). Application of WtCM did not affect mitochondrial area, perimeter or circularity at any time point post-application (**A**, **C**, **E**). Exposure to TgCM did not change any of these parameters at 1h post-application. However, application of TgCM altered area, perimeter and circularity after 8h post-application (**B**, **D**, **F**). Mitochondria became smaller (decreased area and perimeter) and less elongated and more circular (the circularity of a circle is defined as 1), suggesting a disruption of mitochondrial morphology and their functional state. N = 106-145 mitochondria per field of view and time point, 3 independent fields of view per condition, from 2 WtCM and 2 TgCM treated mice. In general, there was a negative although very weak linear correlation between $[Ca^{2+}]_{mit}$ and area, perimeter or circularity 8h after application of TgCM, suggesting a higher calcium concentration in mitochondria and smaller size, and no correlation 1h after application of TgCM or after WtCM application at any time (WtCM area: basal r (Pearson's correlation coefficient) -0.032; *p* 0.0523, 1h r -0.081, *p* 0.107; 8h r -0.103, *p* 0.039. WtCM perimeter: basal r -0.010, *p* 0.838; 1h r -0.077, *p* 0.125; 8h r -0.056, *p* 0.266. WtCM circularity: basal r 0.0146, *p* 0.771; 1h r -0.061, *p* 0.222; 8h r -0.005, *p* 0.920. TgCM area: basal r -0.109, *p* 0.040; 1h r -0.055, *p* 0.306; 8h r -0.109, *p* 0.041; TgCM perimeter: basal r -0.132, *p* 0.013; 1h r -0.075, *p* 0.163; 8h r -0.128, *p* 0.016; TgCM circularity: basal r -0.148, *p* 0.006, 1h r -0.079, *p* 0.136; 8h r -0.136, *p* 0.009).

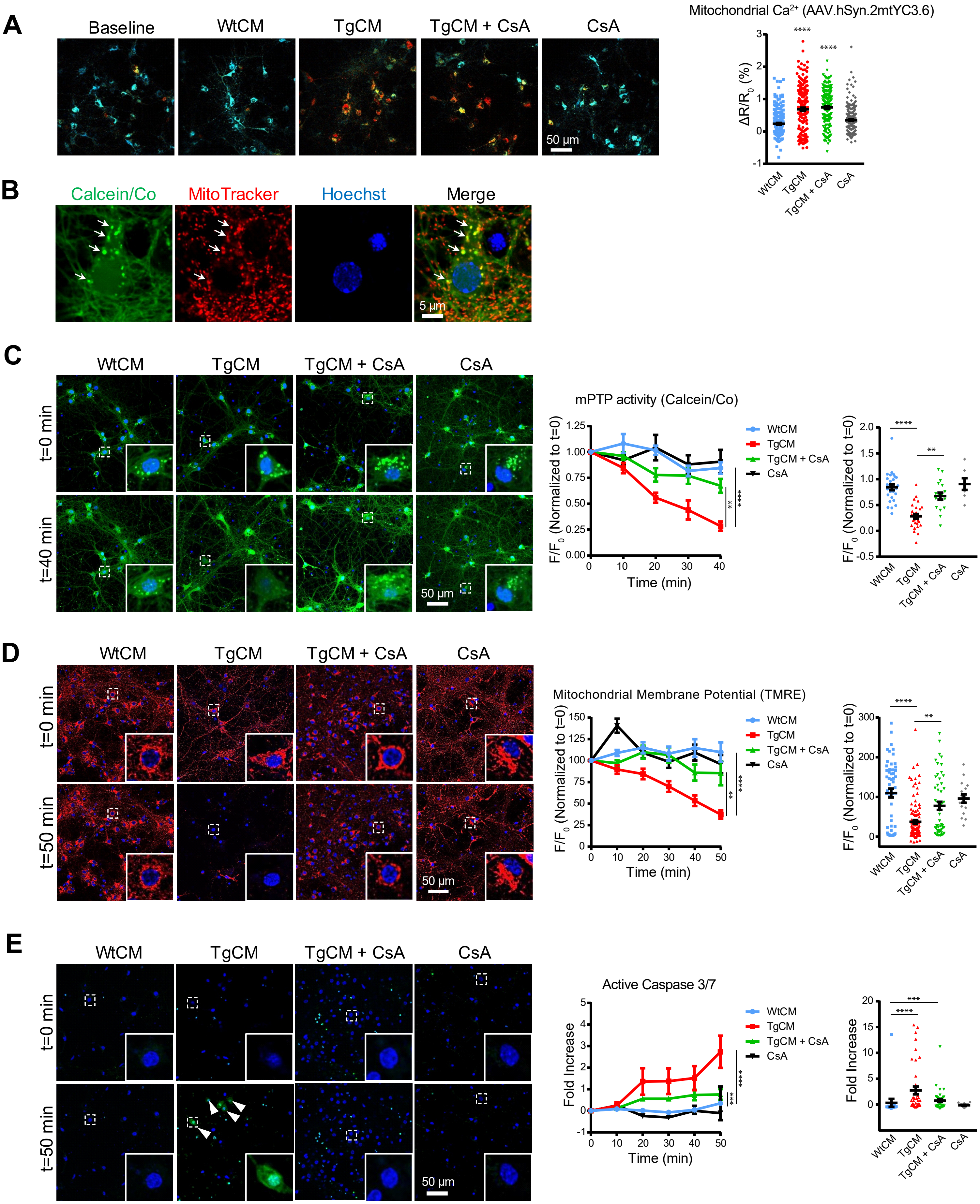
A
Baseline
WtCM
TgCM
TgCM + CsA
CsA
50 μm
Mitochondrial Ca2+ (AAV.hSyn.2mtYC3.6)
ΔR/R0 (%)
****
****
WtCM
TgCM
TgCM + CsA
CsA
B
Calcein/Co
MitoTracker
Hoechst
Merge
5 μm
C
WtCM
TgCM
TgCM + CsA
CsA
t=0 min
t=40 min
50 μm
mPTP activity (Calcein/Co)
F/F0 (Normalized to t=0)
Time (min)
WtCM
TgCM
TgCM + CsA
CsA
**
****
D
WtCM
TgCM
TgCM + CsA
CsA
t=0 min
t=50 min
50 μm
Mitochondrial Membrane Potential (TMRE)
F/F0 (Normalized to t=0)
Time (min)
WtCM
TgCM
TgCM + CsA
CsA
**
****
E
WtCM
TgCM
TgCM + CsA
CsA
t=0 min
t=50 min
50 μm
Active Caspase 3/7
Fold Increase
Time (min)
WtCM
TgCM
TgCM + CsA
CsA
***
****

**Supplementary Figure 8. Related to Figure 7. mPTP is involved in the neurotoxicity caused by soluble Aβ.** TgCM induces mitochondrial $Ca^{2+}$ increase (**A**), mPTP opening (**B**), mitochondrial membrane potential (MMP) decrease (**C**) and activation of caspases (**D**), and most of these effects can be prevented with CsA. **A.** TgCM induces mitochondrial $Ca^{2+}$ increase in primary cortical neurons. Neurons at 12-15 DIV were infected with hsyn.2mtYC3.6 and mitochondrial $Ca^{2+}$ was evaluated with multiphoton microscopy. Pseudocolor images show mitochondrial $Ca^{2+}$ levels in baseline conditions or after application of WtCM, TgCM, TgCM+CsA or CsA alone. CsA was not able to prevent the rise mitochondrial $Ca^{2+}$ driven by TgCM. Scatter dot plot represents the relative ratio increase (ΔR/R0) at the end of the experiment (t=50 min). Error bars represent mean ± SEM. ****$p$<0.0001 (0.23±0.02, n=208 cells WtCM, 0.69±0.04 n=207 cells TgCM, 0.75±0.04 n=187 cells TgCM+CsA, and 0.35±0.03, n=218 cells CsA, 2 independent experiments). **B.** Images show colocalization (arrows) of calcein fluorescence (after cobalt quenching) (green) and mitotracker red. **C.** mPTP activity was directly assessed by calcein/Co method. Primary neurons were loaded with calcein AM and $CoCl_2$ for 15 min and calcein fluorescence quenching was imaged. Images show calcein/Co fluorescence in baseline conditions (t=0 min) and at the end of the experiment (t=40 min). Traces correspond to mean ± SEM fluorescence normalized to the value at t=0 min. CsA prevented the mPTP opening driven by TgCM. ****$p$<0.0001, **$p$<0.005 (0.85±0.06, n=27 cells WtCM, 0.28±0.05 n=28 cells TgCM, 0.67±0.53 n=18 cells TgCM+CsA, and 0.90±0.11, n=7 cells CsA, 2 independent experiments). Scatter dot plot represents the relative fluorescence (normalized to fluorescence t=0 min) at the end of the experiment (t=40 min). Error bars represent mean ± SEM. **D.** MMP was addressed by TMRE in primary neurons exposed to WtCM, TgCM, TgCM+CsA or CsA alone. Images show TMRE fluorescence in baseline conditions (t=0 min) and at the end of the experiment (t=50 min). Fluorescence intensity (normalized to t=0 min) is shown in the traces. Scatter dot plot represents the relative fluorescence intensity (normalized to t=0 min) at the end of the experiment (t=50 min). Error bars represent mean ± SEM. TgCM failed to depolarize mitochondria in presence of CsA. ****$p$<0.0001, **$p$<0.005 (109.8±11.31, n=53 cells WtCM, 37.34±4.65 n=111 cells TgCM, 77.62±10.26 n=51 cells TgCM+CsA, and 95.93±10.57, n=16 cells CsA, 3 independent experiments). **E.** Primary neurons were exposed to either WtCM, TgCM, TgCM+CsA or CsA alone, and caspase 3/7 activation was directly assessed. Pictures show nuclei (blue) and activated caspases (green) fluorescence in baseline conditions (t=0 min) and at the end of the experiment (t=50 min). Traces show mean ± SEM fluorescence fold increase from t=0 min. Scatter dot plot represents the fold increase at the end of the experiment (t=50 min). Error bars represent mean ± SEM. ****$p$<0.0001. Less cells showed activation of caspase 3/7 in presence of CsA after TgCM application (although non-significant) (0.34±0.78, n=18 cells WtCM, 2.74±0.75 n=41 cells TgCM, 0.76±0.25 n=51 cells TgCM+CsA, and -0.11±0.08, n=16 cells CsA from 3 independent experiments).

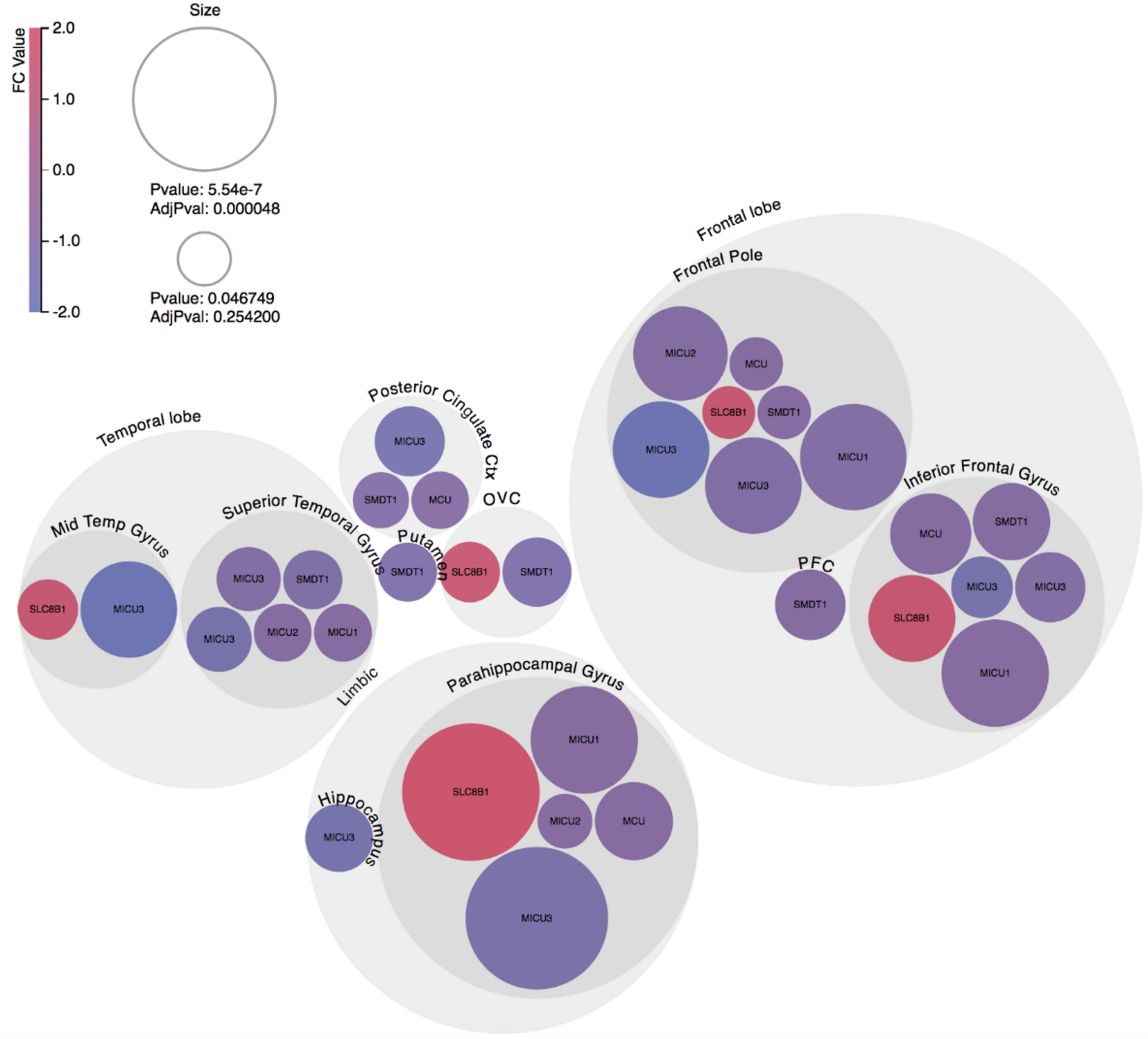


**Supplementary Figure 9. Bubble chart analysis of gene expression changes in the human AD brain.** Microarray and RNA-Seq datasets available at the AMP-AD Knowledge Portal were assessed for differential expression of the following genes involved in mitochondrial $Ca^{2+}$ transport in AD subjects (Braak stages V-VI) *vs.* control individuals (Braak stages 0-I-II): *MCU*, *MCUB*, *MCUR1*, *MICU1*, *MICU2*, *MICU3*, *SMDT1* and *SLC8B1*. Differentially expressed genes were identified at a false discovery rate (FDR) of 25% (listed in Table S1). The size of each bubble is determined by the P value such that highly significant changes are represented by larger bubbles. The smallest bubble corresponds to a P value of 0.0467, and the largest to a P value of $5.54 \times 10^{-7}$. The color of each bubble indicates the direction and magnitude of gene expression fold change (FC), expressed as log2 FC value. Data are plotted by brain region. Genes from each dataset are shown as separate bubbles. Multiple bubbles per gene indicate changes in multiple datasets. OVG, occipital visual cortex; PFC, prefrontal cortex. Four brain regions had both microarray and RNA-Seq data. Data are adjusted for neuronal loss with the pan-neuronal marker MAP2.

| Study | Brain Region | dtype | Contrast | Gene Symbol | logFC | FC | P.Value | P.adjust |
|---|---|---|---|---|---|---|---|---|
| MSBB | Frontal Pole | Microarray | B3-B1 | SLC8B1 | 0.156688 | 1.114725 | 0.04675 | 0.254201 |
| MSBB | Frontal Pole | RNA-Seq | B3-B1 | SMDT1 | -0.0907 | -1.06489 | 0.020704 | 0.248442 |
| MSBB | Frontal Pole | RNA-Seq | B3-B1 | MCU | -0.14279 | -1.10404 | 0.011242 | 0.24451 |
| MSBB | Parahippocampal Gyrus | RNA-Seq | B3-B1 | MICU2 | -0.08512 | -1.06078 | 0.007919 | 0.229649 |
| MSBB | Posterior Cingulate Cortex | Microarray | B3-B1 | SMDT1 | -0.32548 | -1.25308 | 0.04586 | 0.214015 |
| MSBB | Posterior Cingulate Cortex | Microarray | B3-B1 | MCU | -0.13722 | -1.09979 | 0.020531 | 0.198465 |
| MSBB | Superior Temporal Gyrus | RNA-Seq | B3-B1 | MICU2 | -0.1631 | -1.11969 | 0.004483 | 0.19501 |
| MSBB | Superior Temporal Gyrus | RNA-Seq | B3-B1 | MICU1 | -0.09275 | -1.0664 | 0.011635 | 0.193621 |
| MSBB | Putamen | Microarray | B3-B1 | SMDT1 | -0.47166 | -1.3867 | 0.032254 | 0.180625 |
| MSBB | Superior Temporal Gyrus | Microarray | B3-B1 | SMDT1 | -0.38846 | -1.30899 | 0.010235 | 0.180625 |
| MSBB | Middle Temporal Gyrus | Microarray | B3-B1 | SLC8B1 | 0.171672 | 1.126363 | 0.024076 | 0.166012 |
| MSBB | Occipital Visual Cortex | Microarray | B3-B1 | SLC8B1 | 0.137877 | 1.100285 | 0.030261 | 0.166012 |
| MSBB | Inferior Frontal Gyrus | Microarray | B3-B1 | MICU3 | -0.65164 | -1.57096 | 0.046424 | 0.155342 |
| MSBB | Superior Temporal Gyrus | RNA-Seq | B3-B1 | MICU3 | -0.24986 | -1.18909 | 0.030893 | 0.133158 |
| MSBB | Superior Temporal Gyrus | Microarray | B3-B1 | MICU3 | -0.65925 | -1.57926 | 0.012897 | 0.124667 |
| MSBB | Hippocampus | Microarray | B3-B1 | MICU3 | -0.69333 | -1.61701 | 0.028913 | 0.104811 |
| MSBB | Occipital Visual Cortex | Microarray | B3-B1 | SMDT1 | -0.49052 | -1.40495 | 0.010331 | 0.096427 |
| MSBB | Inferior Frontal Gyrus | RNA-Seq | B3-B1 | MICU3 | -0.21943 | -1.16428 | 0.007237 | 0.089941 |
| MSBB | Posterior Cingulate Cortex | Microarray | B3-B1 | MICU3 | -0.71788 | -1.64477 | 0.019315 | 0.089941 |
| ROSMAP | Dorsolateral Prefrontal Cortex | RNA-Seq | B3-B1 | SMDT1 | -0.13563 | -1.09857 | 0.003131 | 0.087666 |
| MSBB | Inferior Frontal Gyrus | RNA-Seq | B3-B1 | SMDT1 | -0.12827 | -1.09299 | 0.000635 | 0.053319 |
| MSBB | Parahippocampal Gyrus | RNA-Seq | B3-B1 | MCU | -0.08001 | -1.05702 | 0.001151 | 0.050077 |
| MSBB | Inferior Frontal Gyrus | RNA-Seq | B3-B1 | MCU | -0.1363 | -1.09909 | 0.000451 | 0.039272 |
| MSBB | Inferior Frontal Gyrus | RNA-Seq | B3-B1 | SLC8B1 | 0.248295 | 1.187803 | 0.000561 | 0.024387 |
| MSBB | Frontal Pole | RNA-Seq | B3-B1 | MICU2 | -0.17759 | -1.13099 | 0.000145 | 0.012633 |
| MSBB | Frontal Pole | Microarray | B3-B1 | MICU3 | -0.92525 | -1.89902 | 0.000268 | 0.01032 |
| MSBB | Frontal Pole | RNA-Seq | B3-B1 | MICU3 | -0.3387 | -1.26462 | 0.001046 | 0.01032 |
| MSBB | Middle Temporal Gyrus | Microarray | B3-B1 | MICU3 | -0.95353 | -1.93661 | 0.001779 | 0.01032 |
| MSBB | Frontal Pole | RNA-Seq | B3-B1 | MICU1 | -0.22081 | -1.16539 | 0.000136 | 0.003939 |
| MSBB | Inferior Frontal Gyrus | RNA-Seq | B3-B1 | MICU1 | -0.11186 | -1.08062 | 8.15E-05 | 0.003543 |
| MSBB | Parahippocampal Gyrus | RNA-Seq | B3-B1 | MICU1 | -0.03251 | -1.02279 | 6.57E-05 | 0.003543 |
| MSBB | Parahippocampal Gyrus | RNA-Seq | B3-B1 | SLC8B1 | 0.43759 | 1.35434 | 1.10E-06 | 9.60E-05 |
| MSBB | Parahippocampal Gyrus | RNA-Seq | B3-B1 | MICU3 | -0.55772 | -1.47194 | 5.54E-07 | 4.82E-05 |

**Supplementary Table 1. Related to Supplementary Figure 9. Analysis of mitochondria $Ca^{2+}$ homeostasis genes in human datasets.** The details (study, brain region, etc.) of the 25 human datasets downloaded from the AMP-AD knowledge portal are included. Datasets used include microarray and RNA-seq. These datasets span 19 brain regions. Within these 25 datasets, comparisons between AD subjects (B3, Braak stages V-VI) *vs.* control individuals (B1, Braak stages 0-I-II) were made for *MCU*, *MCUB*, *MCUR1*, *MICU1*, *MICU2*, *MICU3*, *SMDT1* and *SLC8B1* genes. Fold-change (FC) values, unadjusted *P* values and *P* values adjusted for multiple comparisons (Benjamini-Hochberg) are given.

| Study | Brain region | dtype | Contrast | GeneSymbol | logFC | FC | P.Value | P.adj |
|---|---|---|---|---|---|---|---|---|
| MSBB | Inferior Frontal Gyrus | RNA-Seq | C3-C0 | MCU | -0.11256 | -1.08114 | 0.00258 | 0.094932 |
| MSBB | Parahippocampal Gyrus | RNA-Seq | C3-C0 | MCU | -0.05291 | -1.03735 | 0.003114 | 0.094932 |
| MSBB | Prefrontal Cortex | Microarray | C3-C0 | MCU | -0.18069 | -1.13343 | 0.009821 | 0.094932 |
| MSBB | Anterior Cingulate | Microarray | C3-C0 | MCUR1 | 0.517809 | 1.431779 | 0.002487 | 0.024037 |
| ROSMAP | Dorsolateral Prefrontal Cortex | Microarray | C3-C0 | MCUR1 | 0.102631 | 1.07373 | 0.000346 | 0.01632 |
| MSBB | Dorsolateral Prefrontal Cortex | Microarray | C3-C0 | MCUR1 | 0.40757 | 1.32645 | 0.013429 | 0.146042 |
| MSBB | Inferior Temporal Gyrus | Microarray | C3-C0 | MCUR1 | 0.529005 | 1.442934 | 0.001126 | 0.01632 |
| ROSMAP | Dorsolateral Prefrontal Cortex | RNA-Seq | C3-C0 | MICU1 | -0.06547 | -1.04642 | 0.003466 | 0.075396 |
| MSBB | Frontal Pole | RNA-Seq | C3-C0 | MICU1 | -0.13625 | -1.09904 | 0.012327 | 0.178735 |
| MSBB | Inferior Frontal Gyrus | RNA-Seq | C3-C0 | MICU1 | -0.09279 | -1.06643 | 0.000578 | 0.016766 |
| MSBB | Parahippocampal Gyrus | RNA-Seq | C3-C0 | MICU1 | -0.04191 | -1.02948 | 6.01E-06 | 0.000523 |
| MSBB | Superior Temporal Gyrus | RNA-Seq | C3-C0 | MICU1 | -0.06637 | -1.04708 | 0.015284 | 0.132968 |
| MSBB | Superior Temporal Gyrus | Microarray | C3-C0 | MICU2 | -0.67485 | -1.59643 | 0.002138 | 0.186011 |
| MSBB | Dorsolateral Prefrontal Cortex | Microarray | C3-C0 | MICU3 | -0.59497 | -1.51044 | 0.006029 | 0.131135 |
| MSBB | Frontal Pole | RNA-Seq | C3-C0 | MICU3 | -0.23774 | -1.17914 | 0.015797 | 0.1527 |
| MSBB | Frontal Pole | Microarray | C3-C0 | MICU3 | -0.59382 | -1.50924 | 0.036177 | 0.157372 |
| MSBB | Inferior Frontal Gyrus | RNA-Seq | C3-C0 | MICU3 | -0.1954 | -1.14504 | 0.01545 | 0.1527 |
| MSBB | Middle Temporal Gyrus | Microarray | C3-C0 | MICU3 | -0.59192 | -1.50725 | 0.040232 | 0.1527 |
| MSBB | Parahippocampal Gyrus | RNA-Seq | C3-C0 | MICU3 | -0.51129 | -1.42533 | 1.49E-06 | 0.00013 |
| MSBB | Superior Temporal Gyrus | Microarray | C3-C0 | MICU3 | -0.83028 | -1.77803 | 0.003192 | 0.092577 |
| MSBB | Superior Temporal Gyrus | RNA-Seq | C3-C0 | MICU3 | -0.22497 | -1.16876 | 0.034902 | 0.1527 |
| MSBB | Inferior Frontal Gyrus | RNA-Seq | C3-C0 | SLC8B1 | 0.230494 | 1.173237 | 0.000702 | 0.030548 |
| MSBB | Parahippocampal Gyrus | RNA-Seq | C3-C0 | SLC8B1 | 0.374231 | 1.296149 | 2.50E-05 | 0.002176 |
| MSBB | Anterior Cingulate | Microarray | C3-C0 | SMDT1 | 0.41651 | 1.334695 | 0.008228 | 0.115192 |
| ROSMAP | Dorsolateral Prefrontal Cortex | RNA-Seq | C3-C0 | SMDT1 | -0.11406 | -1.08227 | 0.000389 | 0.021761 |
| MSBB | Inferior Frontal Gyrus | RNA-Seq | C3-C0 | SMDT1 | -0.09666 | -1.06929 | 0.00599 | 0.167722 |
| MSBB | Inferior Temporal Gyrus | Microarray | C3-C0 | SMDT1 | 0.512005 | 1.426031 | 0.027795 | 0.174205 |
| MSBB | Posterior Cingulate Cortex | Microarray | C3-C0 | SMDT1 | -0.35936 | -1.28286 | 0.031108 | 0.174205 |

**Supplementary Table 2. Related to Supplementary Figure 9. CERAD analysis of mitochondria $Ca^{2+}$ homeostasis genes in human datasets.** Within the 25 human datasets downloaded from the AMP-AD knowledge portal, comparison between frequent neuritic plaques (C3) *vs*. absent neuritic plaques (C0) were made for *MCU*, *MCUB*, *MCUR1*, *MICU1*, *MICU2*, *MICU3*, *SMDT1* and *SLC8B1* genes. Fold-change (FC) values, unadjusted *P* values and *P* values adjusted for multiple comparisons (Benjamini-Hochberg) are given.
