# Supplementary material for: Increased mitochondrial calcium levels associated with neuronal death in a mouse model of Alzheimer’s disease
Source: Nat Commun. 2020 May 1;11:2146. doi: 10.1038/s41467-020-16074-2 (PMC7195480; doi:10.1038/s41467-020-16074-2)
Supplement: Supplementary file 3 — Reporting Summary [file 41467_2020_16074_MOESM3_ESM.pdf]

nature research

Corresponding author(s): Brian J. Bacskai
Last updated by author(s): 03/16/2020

Reporting Summary

Nature Research wishes to improve the reproducibility of the work that we publish. This form provides structure for consistency and transparency in reporting. For further information on Nature Research policies, see Authors & Referees and the Editorial Policy Checklist .

Statistics

For all statistical analyses, confirm that the following items are present in the figure legend, table legend, main text, or Methods section.

n/a	Confirmed	
☐	☒	The exact sample size (*n*) for each experimental group/condition, given as a discrete number and unit of measurement
☐	☒	A statement on whether measurements were taken from distinct samples or whether the same sample was measured repeatedly
☐	☒	The statistical test(s) used AND whether they are one- or two-sided<br>*Only common tests should be described solely by name; describe more complex techniques in the Methods section.*
☒	☐	A description of all covariates tested
☐	☒	A description of any assumptions or corrections, such as tests of normality and adjustment for multiple comparisons
☐	☒	A full description of the statistical parameters including central tendency (e.g. means) or other basic estimates (e.g. regression coefficient) AND variation (e.g. standard deviation) or associated estimates of uncertainty (e.g. confidence intervals)
☒	☐	For null hypothesis testing, the test statistic (e.g. F, t, r) with confidence intervals, effect sizes, degrees of freedom and P value noted<br>*Give P values as exact values whenever suitable.*
☒	☐	For Bayesian analysis, information on the choice of priors and Markov chain Monte Carlo settings
☒	☐	For hierarchical and complex designs, identification of the appropriate level for tests and full reporting of outcomes
☒	☐	Estimates of effect sizes (e.g. Cohen's d, Pearson's r), indicating how they were calculated

Our web collection on statistics for biologists contains articles on many of the points above.

Software and code

Policy information about availability of computer code

Data collection	Commercial microscope softward was used for data collection.
Data analysis	Microscopy images were prepared using ImageJ (Fiji). Matlab, SAS and GraphPad 5.0 were used for data analysis.

For manuscripts utilizing custom algorithms or software that are central to the research but not yet described in published literature, software must be made available to editors/reviewers. We strongly encourage code deposition in a community repository (e.g. GitHub). See the Nature Research guidelines for submitting code & software for further information.

Data

Policy information about availability of data

All manuscripts must include a data availability statement. This statement should provide the following information, where applicable:

- Accession codes, unique identifiers, or web links for publicly available datasets
- A list of figures that have associated raw data
- A description of any restrictions on data availability

The data that support the findings of this study are available from the corresponding author upon reasonable request.

Field-specific reporting

Please select the one below that is the best fit for your research. If you are not sure, read the appropriate sections before making your selection.

☒ Life sciences ☐ Behavioural & social sciences ☐ Ecological, evolutionary & environmental sciences

For a reference copy of the document with all sections, see nature.com/documents/nr-reporting-summary-flat.pdf

Life sciences study design

All studies must disclose on these points even when the disclosure is negative.

Sample size	No statistical methods were used to predetermine sample size. Sample size per group was determined from previous publications with similar methodologies.
Data exclusions	When injection of the AAV or craniotomy was not successful, we excluded those mice
Replication	For each animal experiment, at least three volumes were analyzed per animal, and at least 3 animals were used per condition. Replication of experiments was successful.
Randomization	All volumes analyzed were randomly selected. For conditioned media experiments, mice were randomized into either WtCM, TgCM or depleted TgCM.
Blinding	Investigators were blinded to genotype and for data analysis

Reporting for specific materials, systems and methods

We require information from authors about some types of materials, experimental systems and methods used in many studies. Here, indicate whether each material, system or method listed is relevant to your study. If you are not sure if a list item applies to your research, read the appropriate section before selecting a response.

Materials & experimental systems

n/a	Involved in the study	
☐	☒	Antibodies
☐	☒	Eukaryotic cell lines
☒	☐	Palaeontology
☐	☒	Animals and other organisms
☒	☐	Human research participants
☒	☐	Clinical data

Methods

n/a	Involved in the study	
☒	☐	ChIP-seq
☒	☐	Flow cytometry
☒	☐	MRI-based neuroimaging

Antibodies

Antibodies used

mouse antibody 6E10 (Purified anti-β-Amyloid, 1-16 antibody BioLegend Cat# 803004, RRID: AB_2715854)
rabbit anti-MCU (1:1000, Sigma-Aldrich Cat# HPA016480, RRID:AB_2071893),
rabbit anti-MiCU1 (1:1000, Sigma-Aldrich Cat# HPA037480, RRID:AB_10696934),
rabbit anti-EFHA1 (MiCU2) (1:500, Abcam Cat# ab101465, RRID:AB_10711219),
rabbit anti-SLC24A6 (NCLX) (1:250, Sigma-Aldrich Cat# SAB2102181, RRID:AB_10606696),
mouse anti-VDAC1/porin (1:1000, Abcam Cat# ab14734, RRID:AB_443084),
mouse anti-β-tubulin I (1: 10,000, Sigma-Aldrich Cat# T7816, RRID:AB_261770),
antibody against GFP (chicken anti-GFP IgY, 1:500, Aves Labs Cat# GFP-1020, RRID:AB_2307313)
HSP60 (rabbit anti-HSP60, 1:500, Abcam Cat# ab46798, RRID:AB_881444),
NeuN (mouse anti-NeuN, 1:500 Millipore Cat# MAB377, RRID:AB_2298772),
GS (rabbit anti-Glutamine Synthetase, 1:500, Abcam Cat# ab73593, RRID:AB_2247588),
Alexa fluor 488-conjugated goat anti-chicken IgG antibody (1:500, Molecular Probes Cat# A-11039, RRID:AB_142924),
Cy3-conjugated goat anti-rabbit IgG antibody (1:1000, Abcam Cat# ab6939, RRID:AB_955021)
Cy3-conjugated goat anti-mouse IgG antibody (1:1000, Abcam Cat# ab97035, RRID:AB_10680176),
Cy5-conjugated goat anti-rabbit IgG antibody (1:1000, Jackson ImmunoResearch Labs Cat# 111-175-144, RRID:AB_2338013)

Validation

Antibodies were purchased from commercial sources. When possible, commercial antibodies with specific publications of previous use were purchased.
Validation on manufacturer's website. For immunostaining, positive and negative controls also allowed for validation of antibody data, and several dilutions were tested for each antibody.
6E10 (803004) https://www.biolegend.com/en-us/products/purified-anti-beta-amyloid-1-16-antibody-11228
anti-MCU (HPA016480) https://www.sigmaaldrich.com/catalog/product/sigma/hpa016480?lang=en&region=US
anti-MiCU1 (HPA037480) https://www.sigmaaldrich.com/catalog/product/sigma/hpa037480?lang=en&region=US
anti-EFHA1 (ab101465) https://www.abcam.com/micu2-antibody-ab101465.html
anti-SLC24A6 (SAB2102181) https://www.sigmaaldrich.com/catalog/product/sigma/sab2102181?lang=en&region=US
anti-VDAC1 (ab14734) https://www.abcam.com/vdac1-porin-antibody-20b12af2-ab14734.html
anti-β-tubulin I (T7816) https://www.sigmaaldrich.com/catalog/product/sigma/t7816?lang=en&region=US
anti-GFP (GFP-1020) https://www.aveslab.com/collections/epitope-tag-6xhis-beta-gal-actin-and-gfp-antibodies/products/green-

fluorescent-protein-gfp-antibody
HSP60 (ab46798) https://www.abcam.com/hsp60-antibody-ab46798.html
NeuN (MAB377) https://www.emdmillipore.com/US/en/product/Anti-NeuN-Antibody-clone-A60,MM_NF-MAB377?ReferrerURL=https%3A%2F%2Fwww.google.com%2F&bd=1
GS (ab73593) https://www.abcam.com/glutamine-synthetase-antibody-ab73593.html
Alexa fluor 488 (A-11039) https://www.thermofisher.com/antibody/product/Goat-anti-Chicken-IgY-H-L-Secondary-Antibody-Polyclonal/A-11039
Cy3-conjugated goat anti-rabbit IgG antibody (ab6939) https://www.abcam.com/goat-rabbit-igg-hl-cy3--preadsorbed-ab6939.html
Cy3-conjugated goat anti-mouse IgG antibody (ab97035) https://www.abcam.com/goat-mouse-igg-hl-cy3--preadsorbed-ab97035.html
Cy5-conjugated goat anti-rabbit IgG antibody (111-175-144) https://www.jacksonimmuno.com/catalog/products/111-175-144

Eukaryotic cell lines

Policy information about cell lines

Cell line source(s)	Neuro2a cells were purchased from ATCC
Authentication	This cell line was authenticated by the vendor and our lab
Mycoplasma contamination	This cell line was tested negative for mycoplasma contamination
Commonly misidentified lines (See ICLAC register)	No misidentified cell line was used in this study

Animals and other organisms

Policy information about studies involving animals; ARRIVE guidelines recommended for reporting animal research

Laboratory animals	4-5 months old C57BL/6J mice; 2-3 months old and 8-10 months old APPswe/PSEN1dE9 mice; 4 months old Tg2576 mice
Wild animals	No wild animals were used in this study
Field-collected samples	No field-collected samples were used in this study
Ethics oversight	All animal procedures were performed with the approval of the Massachusetts General Hospital Animal Care and Use Committee and in compliance with the National Institutes of Health Guide for the Care and Use of Laboratory Animals.

Note that full information on the approval of the study protocol must also be provided in the manuscript.
