# Supplementary material for: Increased mitochondrial calcium levels associated with neuronal death in a mouse model of Alzheimer’s disease
Source: Nat Commun. 2020 May 1;11:2146. doi: 10.1038/s41467-020-16074-2 (PMC7195480; doi:10.1038/s41467-020-16074-2)
Supplement: Supplementary file 4 — Description of Additional Supplementary Files [file 41467_2020_16074_MOESM4_ESM.pdf]

Description of Additional Supplementary Files

File Name: Supplementary Movie 1

Description: **Related to Figure 1. Validation of AAV.hSyn.2mtYC3.6 in vivo. KCl** was topically applied to a C57BL/6J mouse brain expressing AAV.hSyn.2mtYC3.6 in the cortex. KCl increased mitochondrial Ca2+ in the recorded neurons.
